# Expression Analysis of Circulating microRNAs in Saliva and Plasma for the Identification of Clinically Relevant Biomarkers for Oral Squamous Cell Carcinoma and Oral Potentially Malignant Disorders

**DOI:** 10.3390/cancers16172990

**Published:** 2024-08-28

**Authors:** Federica Rocchetti, Gianluca Tenore, Federica Macali, Teresa Vicidomini, Gian Marco Podda, Paolo Junior Fantozzi, Valentina Silvestri, Virginia Porzio, Virginia Valentini, Laura Ottini, Antonio Giovanni Richetta, Valentino Valentini, Marco Della Monaca, Camilla Grenga, Antonella Polimeni, Umberto Romeo

**Affiliations:** 1 Department of Oral and Maxillofacial Sciences, Sapienza University of Rome, 00161 Rome, Italy; federica.rocchetti@uniroma1.it (F.R.); macali.1630241@studenti.uniroma1.it (F.M.); vicidomini.1859365@studenti.uniroma1.it (T.V.); gianmarco.podda@uniroma1.it (G.M.P.); paolojunior.fantozzi@uniroma1.it (P.J.F.); valentino.valentini@uniroma1.it (V.V.); marco.dellamonaca@uniroma1.it (M.D.M.); camilla.grenga@uniroma1.it (C.G.); antonella.polimeni@uniroma1.it (A.P.); umberto.romeo@uniroma1.it (U.R.); 2 Department of Molecular Medicine, Sapienza University of Rome, 00161 Rome, Italy; valentina.silvestri@uniroma1.it (V.S.); virginia.porzio@uniroma1.it (V.P.); virginia.valentini@uniroma1.it (V.V.); laura.ottini@uniroma1.it (L.O.); 3 Department of Internal Medicine and Medical Specialties, Sapienza University of Rome, 00161 Rome, Italy; antonio.richetta@uniroma1.it

**Keywords:** liquid biopsy, oral squamous cell carcinoma, oral potentially malignant disorders, microRNAs, non-invasive biomarkers

## Abstract

**Simple Summary:**

In recent years, liquid biopsy has been introduced as a new method for the detection and management of cancer, but the studies on oral squamous cell carcinoma (OSCC) are lacking conclusive evidence. The aim of this study was to evaluate the expression of six circulating salivary and plasmatic miRNAs (-21, -31, -138, -145, -184, and -424) as diagnostic biomarkers in patients affected by OSCC and by oral potentially malignant disorders (OPMDs). Our results showed that liquid biopsy from saliva may be a useful tool for the identification of these biomarkers; in particular, miR-138 and miR-424 showed decreased expression levels in saliva samples in OSCC and OPMD patients compared to healthy controls. The introduction of liquid biopsy in daily clinical practice could revolutionize the approach to oral lesions by allowing the mass screening, stratification and monitoring of patients at risk, the monitoring of the response to treatment and the early identification of any recurrences.

**Abstract:**

This study aims to evaluate the expression of salivary and plasmatic miRNAs as diagnostic biomarkers in patients with oral squamous cell carcinoma (OSCC) and oral potentially malignant disorders (OPMDs). A total of 25 patients were divided into three groups, according to their diagnosis: OSCC patients (*n* = 14); OPMDs patients (*n* = 6); and healthy controls (*n* = 5). At the time at diagnosis/enrolment, patients underwent salivary and plasmatic collection. The expression of miRNA -21, -31, -138, -145, -184, and -424 were evaluated by real-time PCR. An F-test and ANOVA test were performed to evaluate the miRNA levels (significance at *p* < 0.05). By comparing miRNA expression levels from saliva, a statistically significant difference emerged in the expression of miR-138 and miR-424 between the three groups (*p* < 0.05). In particular, these two miRNAs showed decreased expression levels in saliva samples from OSCC and OPMD patients compared to those from healthy controls. On the other hand, miRNA expression levels in plasma were low in all the groups, and no statistically significant differences were found. Overall, our results showed that liquid biopsy from saliva may be a useful tool for the identification of diagnostic molecular biomarkers in OSCC and OPMDs.

## 1. Introduction

Oral squamous cell carcinoma (OSCC) is the most common epithelial malignancy of the oral cavity, representing 90% of oral cancers [1,2].

Despite improvements in prevention, diagnosis, and therapeutic strategies, OSCC remains a serious public health concern in several countries including Italy, with an estimated global incidence of more than 300,000 cases per year, about 180,000 deaths per year, and a 5-year relative survival rate of 65% [3,4]. Although the oral cavity is an easily accessible anatomical site, OSCC is often diagnosed at an advanced stage, partly due to the low oral cancer awareness of the population, difficulties in seeking medical assistance (mainly in less developed countries), and to the oftentimes inappropriate management of pre-cancer conditions. As a consequence, OSCC patients require a multimodal therapeutic approach leading to serious physical, functional, aesthetic, and psychosocial complications [5,6].

OSCC is a multifactorial disease with heavy tobacco consumption (including smoked tobacco and areca nut consumption) and heavy alcohol abuse being the major risk factors. Other risk factors include persistent infection of high-risk oncogenic human papillomaviruses (HPVs), poor oral hygiene, nutritional deficiencies, chronic mechanical trauma, and inheritable cancer syndromes (i.e. Cowden syndrome, Bloom syndrome, dyskeratosis congenita, etc.) [7,8]. OSCC may arise “de novo” or from oral potentially malignant disorders (OPMDs), a heterogeneous spectrum of conditions of the oral mucosa, such as oral leukoplakia, proliferative verrucous leukoplakia (PVL), erythroplakia, oral lichen planus (OLP), and oral submucous fibrosis, defined by the WHO as any oral mucosal abnormality that is associated with a statistically increased risk of developing oral cancer [9,10,11,12].

To date, clinical examination of the oral cavity and surgical biopsy of the suspected lesion, followed by histopathological analysis, represent the gold standard in detecting OSCC and defining the histological features of OPMDs [13,14].

However, tissue biopsy has several limitations mainly related to its local invasiveness, the need for trained oral medicine/oral surgery providers, and the lack of ability to determine the intratumor heterogeneity [15,16,17].

It is well documented that carcinogenesis is a complicated, multifactorial process in which the tumor microenvironment, including heterogeneity in genetics, epigenetics, and/or phenotypic changes, plays a quintessential role in local and distant metastases, resistance to therapy, prognosis of the patient, and therefore overall cancer survivorship [18,19,20,21].

Based on these premises, the identification of molecular biomarkers expressed in patients with OSCC and OPMDs might have a crucial impact in early diagnosis and prognosis and provide new insights into individualized precision medicine to improve clinical decision-making in these patients [22].

In this context, liquid biopsy, defined as a non-invasive or minimally invasive procedure involving the molecular analysis of body fluids, such as blood and saliva, appears as a powerful tool for early disease diagnosis and progression and response-to-treatment monitoring [23,24]. Circulating microRNAs (miRNAs) are encouraging potential biomarkers associated with cancer onset and progression, malignant potential, and response to treatment, as they are extremely stable in body fluids [25]. Recent studies on circulating miRNAs in plasma, serum, saliva, and other body fluids showed that miRNAs secreted by certain cell types may not only have local effects but may also act at distant sites [26,27]. Recently, circulating miRNAs have been demonstrated to show differential levels in the body fluids of oral cancer patients compared to healthy individuals, suggesting their potential to be used as oral cancer biomarkers [28]. 

Oncogenic miR-21, miR-31, and miR-184 were found significantly overexpressed in both saliva and plasma samples of OSCC cases as compared with controls [17,29,30]. 

On the other hand, a lower expression of miR-138 and miR-145, two tumor suppressor miRNAs, was found in plasma and saliva samplesof OSCC patients compared to healthy controls [31,32,33]. Interestingly, miR-424 was previously described as both an oncogenic and tumor suppressor miR, with an increase in miR-424 expression reported in plasma samples and a decrease in miR-424 expression in saliva samples from OSCC patients compared with controls [32,34]. Overall, previous studies indicated that these miRNAs may have potential applications as biomarkers for the early diagnosis of OSCC, deserving further investigation [17,35].

The aim of this study was to evaluate the expression of six circulating salivary and plasmatic miRNAs (-21, -31, -138, -145, -184 and -424) as diagnostic biomarkers in patients with OSCC and OPMDs.

## 2. Materials and Methods

### 2.1. Patients’ Cohort and Sample Collection

This was a prospective cohort study of patients enrolled consecutively at the Oral Medicine Unit, Department of Oral and Maxillofacial Sciences, Sapienza University of Rome, Policlinico Umberto I University Hospital of Rome, from October 2022 to January 2024. The inclusion criteria were patients (1) with a histologically confirmed diagnosis of OSCC or OPMD; 2) age ≥ 18 years. The exclusion criteria were patients (1) who were not subjected to histopathological examination; (2) with age < 18 years; (3) patients with periodontal diseases; (4) patients with other malignancies (non-OSCC); (5) patients with diabetes; (6) patients who had undergone an organ transplant; (7) patients with a positive history of chronic viral diseases; and (8) pregnant or lactating women [36,37].

For the analysis of tobacco use, smoking habit was recorded as positive for both active and ex-smokers ≤ 10 years of cessation [38,39]. To analyze alcohol use, the consumption of at least one alcohol unit per day (1 unit = 8–10 g of ethanol = 1 glass of wine = 1/4 L of beer = 1 measure of liqueur) was considered alcohol exposure, according to Pentenero et al. [40].

For the purpose of the study, patients were divided into three groups according to their diagnosis: group 1 (*n* = 14), OSCC patients; group 2 (*n* = 6), OPMD with mild or moderate dysplasia patients; and group 3 (*n* = 5), healthy individuals.

All 25 patients underwent liquid biopsy from saliva and blood samples at time at diagnosis/enrolment. Patients who underwent liquid biopsy did not drink liquids, eat, or consume chewing gum in the 90 minutes prior to the biopsy, following the manufacturer’s protocol. Saliva samples were collected by spitting into Saliva RNA Collection and Preservation Devices (Norgen Biotek Corp., Thorold, ON, Canada). After collection, a preservation solution was added and mixed with the required volume of saliva (2 mL) to preserve the RNA for up to 2 months at room temperature (16–24 °C), according to the manufacturer’s instructions. Blood samples were collected in Blood RNA cfDNA/cf-RNA 10 mL Preservative Tubes, containing preservation reagent (Norgen Biotek Corp., Thorold, ON, Canada), following the manufacturer’s protocol. The required volume of blood (8.4 mL) was stored at room temperature (16–24 °C) and delivered within 48 h to the laboratory for molecular analysis. The study was approved by the Local Ethical Committee of the Policlinico Umberto I of Rome with the reference No. 0186/2022. All procedures performed in this study were in accordance with ethical standards and/or the national research committee and the 2002 Helsinki Declaration and its later amendments or comparable ethical standards. Written informed consent was obtained from all participants.

### 2.2. RNA Extraction and miRNA Expression Analysis

RNA extraction from saliva was performed using the saliva/swab RNA purification kit (Norgen Biotek Corp., Thorold, ON, Canada), following the supplier’s protocol. RNA extraction from plasma was performed using the plasma/serum RNA purification mini kit (Norgen Biotek Corp., Thorold, ON, Canada) following the supplier’s protocol. The RNA concentration of each sample was assessed using a Qubit 2.0 Fluorometer. The expression profile of the six miRNAs selected for their known role in cancer, including miR-21, miR-31, miR-138, miR-145, miR-184 and miR-424, was analysed in all samples by quantitative Real-Time PCR (qPCR) using commercially available TaqMan Advanced miRNA Assays (Thermo Fisher Scientific, Waltham, MA, USA). Reverse transcription was performed using the TaqMan Advanced miRNA cDNA Synthesis Kit (Thermo Fisher Scientific, Waltham, MA, USA) following the manufacturer’s protocol, and qRT-PCR was carried out on a 7500 Fast Real-Time PCR platform (Thermo Fisher Scientific, Waltham, MA, USA). This method allows to quantify mature miRNAs with high sensitivity and specificity, starting from 10 ng of total RNA input. Fluorescence is monitored during 40 amplification cycles. For each target miRNA, the Ct parameter (the cycle at which fluorescence exceeds the threshold level) was normalized using miR-16 as an endogenous control and expressed as ΔCt (Ct target–Ct endogenous control). The relative amount of target miRNA expression in each sample was calculated using the 2^-ΔCt^ method and represented graphically in logarithmic form. Each experiment was repeated in triplicate, and at least one negative control was included for each reaction.

### 2.3. Statistical Analysis

Statistical analysis was performed using StatSoft, Inc. STATISTICA^®^ version 12, Helsinki, Finland. An F- test was carried out to evaluate the homogeneity between the variances of the groups to be compared, which in this case confirmed the homoscedasticity. Subsequently, for each miRNA and for each group, a comparison was made between quantitative distributions through the analysis of variance (One-Way ANOVA) with the hypothesis of homoscedasticity. Additionally, the One-Way ANOVA test was used to analyze the association between the concentrations of miRNAs in G1 with some variables, such as patient age and gender, smoking habit, drinking habit, and OSCC stage and localization. The significance threshold was set at *p* < 0.05.

## 3. Results

### 3.1. Clinical–Pathologic Characteristics of the Patients‘Cohort

The patients’ clinical data are summarized in Table 1. Overall, there were 14/25 (56.0%) patients with OSCC (*n* = 8 males; 57.1%; *n* = 6 females; 42.9%), 6/25 (24.0%) patients with OPMDs (*n* = 3 males; 50.0%; *n* = 3 females, 50.0%), and 5/25 (*n* = 2 males; 40%; *n* = 3 females; 60.0%) controls. The overall entire cohort median age was 68.08 years (range: 48–84), with the OSCC cohort having a median age of 70.2 years (range: 52–84), and the OPMD cohort having a median age of 67.6 years (range: 57–79) and the healthy subjects cohort having a median age of 62.6 years (range: 48–80). 

Overall, 5 out of 25 patients (20.0%) had a personal history of any cancer (*n* = 3/14 OSCC; 21.4%); (*n* = 2/6 OPMDs; 33.3%), whereas when social history was considered, 10/25 (40.0%) patients were current or former tobacco smokers (*n* = 6/14 OSCC; 42.9%) and 2/25 were alcohol consumers (*n* = 2/14 OSCC; 14.3%). 

In G1, when the OSCC location was considered, 8/14 (57.1%) had a diagnosis of tongue OSCC, followed by the alveolar ridge/gingiva (*n* = 3, 21.4%), buccal mucosa (*n* = 2, 14.3%), and floor of the mouth (*n* = 1, 7.1%). All cases of OSCC were moderately differentiated malignancies (grade 2, G2). When the TNM classification was considered, most of the patients had stage IV (*n* = 6, 42.9%) oral cancer, followed by four patients affected by stage (28.6%), three patients with stage III (21.4%) and one patient with stage II (7.1%). According to the OSCC stage, patients affected by stage I/II were treated by surgical tumor resection while patients with stage III/IV were treated by surgical tumor resection and radiotherapy.

In G2, three patients (50.0%) were affected by OLP with mild dysplasia, two patients (30.0%) by PVL (one with mild and one with moderate dysplasia), and one patient (10.0%) by oral lichenoid lesions (OLL) with mild dysplasia. When the OPMD location was considered, 5/6 (83.3%) patients had lesions localized on the buccal mucosa, whereas in 2/6 (33.3%) patients, the lesions were located on the tongue. In terms of treatment regimens, patients affected by OLP were treated with topical (clobetasol 0.05% oral gel BID) or systemic steroids until resolution [41]; patients affected by PVL were treated by CO_2_ laser ablation or surgical excision by scalpel and subsequent follow-up [42]; and the patients affected by OLL were treated by surgical resection and the removal and replacement of dental material [12].

### 3.2. miRNA Expression Analysis

Overall, the quality and quantity of RNAs were adequate to perform a molecular analysis for all samples collected.

By comparing miRNA expression levels from saliva samples by the ANOVA test, a statistically significant difference emerged in the expression levels of miR-138 and miR-424 among the three groups (*p* = 0.01 and *p* = 0.04, respectively) (Table 2). 

**Figure 1:**
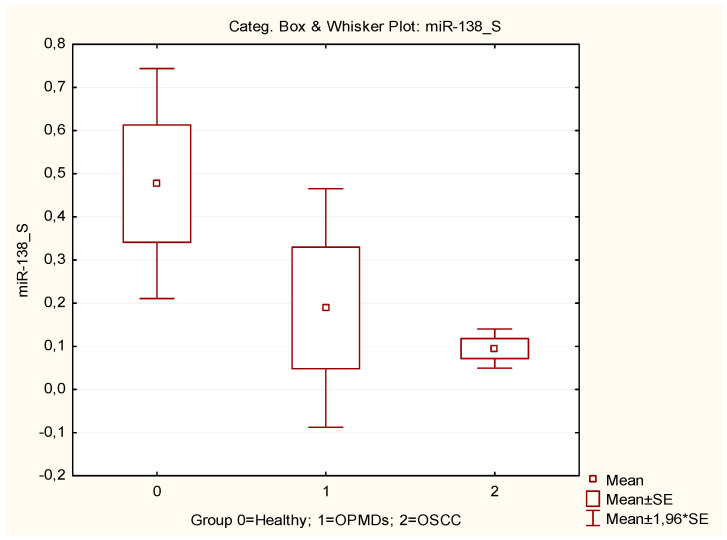
Box plot of miRNA-138 expression levels in saliva samples in OSCC patients, OPMD patients, and healthy controls.

**Figure 2:**
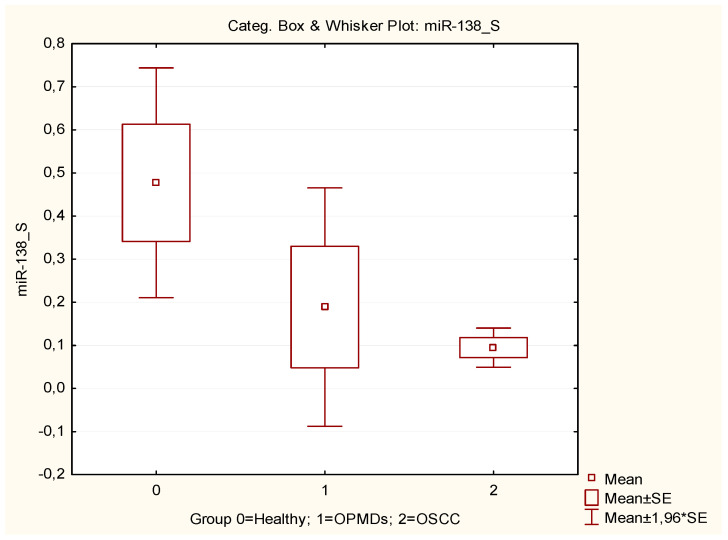
Box plot of miRNA-424 expression levels in saliva samples in OSCC patients, OPMD patients, and healthy controls.

Specifically, both miRNAs showed decreased expression levels in saliva samples in OSCC and OPMD patients compared to healthy controls (Figure 1 and Figure 2).

On the other hand, miRNA expression levels in plasma samples were low in all the three groups, and no statistically significant differences emerged in the expression levels of each miRNA (Table 3).

### 3.3. Associations between miRNA Expression and Clinical–Pathologic Characteristics of OSCC Patients

A significant association was found between the expression of miR-424 in saliva samples and smoking habits (*p* = 0.04) (Table 4). 

**Figure 3:**
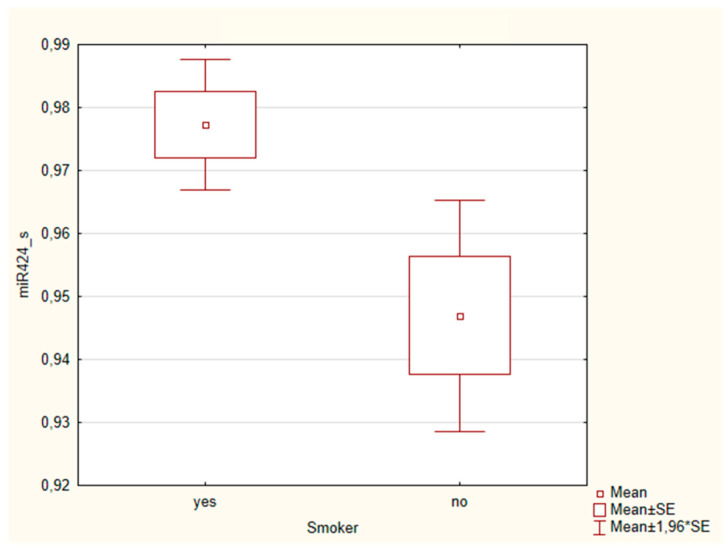
Box plot of the association between salivary miRNA-424 expression levels and smoking habits in OSCC patients.

Specifically, the levels of miR-424 in saliva were higher in non-smoking patients than in smokers (Figure 3).

A significant association was found, also, between the expression of miR-145 in saliva samples and drinking habits (*p* = 0.02) (Table 5). 

**Figure 4:**
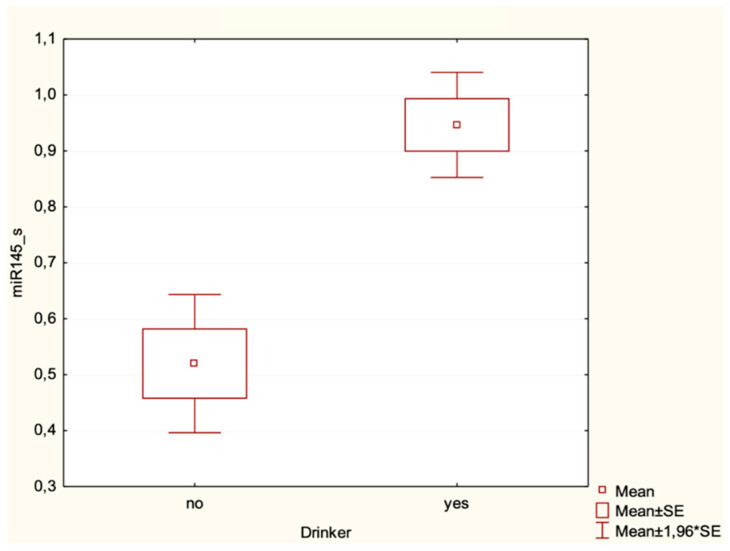
Box plot of the association between salivary miRNA-145_s expression levels and drinking habits in OSCC patients.

Specifically, the levels of miR-145 in saliva were higher in non-drinkers than in alcohol consumers (Figure 4).

There was no significant association between miRNA expression level in saliva and the following characteristics: patient age and gender, OSCC stage, and localization. In plasma samples, no statistically significant association was found between miRNA expression level and the characteristics evaluated.

## 4. Discussion

OSCC is the most common malignancy of the head and neck region [43]. Recently, despite the up-and-coming development of personalized medicine and advances in cancer detection and treatment, the survival rates of OSCC patients have not shown satisfying improvements. There are still many issues that need to be resolved. First of all, due to the lack of an effective and repeatable method to detect OSCC in real time, we do not have enough understanding of the spatial and temporal heterogeneity of OSCC [44,45]. Secondly, when performing a surgical biopsy, while not performing a mapping of the lesion, or an entire excision, the sample only represents a single, localized part of the whole lesion. [46,47]. Another challenge is how to identify OSCC in the early stages, which most of the times is only represented by a subtle, non-symptomatic lesion leading to a late-time detection and subsequent poor prognosis [5,48]. And so far, we cannot monitor the treatment responses and resistance of OSCC in real time by repeated analysis. Liquid biopsy may solve part of the above issues. 

As a matter of fact, liquid biopsy is a non-invasive method whose samples come from body fluids, such as blood, saliva, urine, pleural effusion, cerebrospinal fluid, etc., based on the detection of circulating tumor cells (CTCs), circulating tumor DNA (ctDNA), and circulating tumor RNA (ctRNA), proteins, exosomes, and miRNA [2,45,49].

This study is an attempt to develop an experimental protocol to be easily implemented in clinical settings for the detection of OSCC and OPMDs through the expression analysis of six circulating miRNAs in saliva and plasma samples. 

Blood is the most used body fluid for liquid biopsy, and its use in clinical settings is well documented for different malignant and non-malignant conditions [50]. 

On the other hand, saliva may be a viable alternative, as being less invasive for the patient. Furthermore, in the case of OSCC and OPMDs, this fluid is in direct contact with the lesion site, being able to not only show the systemic expression of biomarkers but also the localized environment adjacent to the tumor itself [51,52,53]. 

The tubes to best preserve the RNA at room temperature and the extraction kits were chosen to have the highest yield in terms of RNA quality and quantity. Overall, the quality and quantity of RNA was adequate to perform a molecular analysis for all the samples collected from saliva and plasma. The Real-Time method to assess microRNA expression was chosen as it allows a sensitive, fast, and accurate analysis. Moreover, the qRT-PCR approach results in a significant time and cost saving (being less expensive than Digital PRC and next-generation sequencing) [54]. On the other hand, to achieve consistent and repeatable results, several steps such as RNA extraction, RNA quantification, and data normalization must be considered [55].

The six microRNAs analyzed (miR-21, -31, -138, -145, -184, and -424) were chosen as they were known from the literature to be involved in OSCC development and progression [56,57]. Circulating miRNAs are easily detected in a small amount of body fluid by conventional PCR or high-throughput platforms, such as microarray and next-generation sequencing [58]. Therefore, several studies have focused on miRNAs as possible candidates for liquid biopsy for early cancer detection [59,60].

Our study included 14 patients with a confirmed histological diagnosis of OSCC and 6 patients with a confirmed histological diagnosis of OPMDs. Overall, our cohort of patients, although limited in number, was representative of the known epidemiologic characteristics of the disease [61].

As a first analysis, a comparison of circulating miR-21, miR-31, miR-138, miR-145, miR-184, and miR-424 expression levels at baseline among OSCC patients, OPMD patients, and healthy controls was performed.

By comparing miRNA expression levels from saliva samples, a statistically significant difference emerged in the expression levels of miR-138 between the three groups. The decreased expression of this miRNA in saliva samples from OSCC and OPMD patients, compared to healthy controls, is in accordance with previous studies highlighting the role of miR-138 as a tumor suppressor [32]. MiR-138 may be able to regulate the biological processes of OSCC via repressing ISG15 expression. ISG15 serves important roles in immune regulation and tumor development [62].

In agreement with our results, miR-138 was shown to be significantly downregulated in tissue and plasma samples of OSCC [63]. It is noteworthy that our study showed that in OPMD lesions, as well as in OSCC cases, miR-138 levels are lower compared to healthy controls, suggesting that this miRNA may be involved in the early phases of carcinogenesis and therefore have clinical relevance as an early diagnostic biomarker.

Notably, our study also provides the first evidence of a possible role of miR-138 as a biomarker in saliva liquid biopsy.

A statistically significant difference also emerged in the expression levels of miR-424 between the three groups in the saliva samples. In particular, a significant decrease in miR-424 expression in saliva samples from OSCC and OPMD patients compared to healthy controls was observed. MiR-424 is an example of miRNA with tissue-specific impacts in carcinogenesis, acting as a tumor suppressor or as an onco-miR based on the tissue. In OSCC, different studies confirmed the upregulation of miR-424 both in OSCC tissues and blood samples, and the overexpression of miR-424 has been associated with poor differentiation, advanced tumor stages, and cervical lymph node involvement [64]. By contrast, in saliva samples, a previous study revealed a downregulation of miR-424 in OSCC patients [34]. The lower expression of salivary miR-424 in OSCC patients suggests a systemic suppression of its production or release from the cells of the oral cavity, probably due to the tumor itself, but the possible advantage of this suppression has not yet been explored [65,66].

MiR-424 has several targets, of which the first studied belong to the E2F family of transcription factors, that are found to be deregulated in several tumor types [64,67]. In oral cancer, miR-424 targets STAT5, involved in cellular aberrant invasion and SOCS2, a suppressor of cytokine signaling [32,68]. The overexpression of miR-424 in oral cancer cells can promote cell migration and invasion [32]. Overall, most of the cancer types in which miR-424 was found to be upregulated, including melanoma, laryngeal and esophageal squamous cell carcinomas, and thyroid cancer, are known to be associated with specific lifestyle factors [64].

An interesting association emerged between smoking history and miR-424 expression in the saliva samples of OSCC patients. At baseline, the levels of miR-424 in saliva samples were higher in non-smoking patients than in ex-smokers and smokers. There are currently no studies in the literature reporting an association between smoking history and miR-424 expression in OSCC. There is evidence of an association between smoking history and miR-424 expression in pulmonary diseases [69,70]. Based on this evidence, we can hypothesize that, as smoking is a known risk factor—not only for pulmonary diseases, but also for oral cancer—miR-424 may be particularly involved in cellular pathways that may functionally interact with environmental risk factors, including smoking.

A further association emerged between alcohol consumption and miR-145 expression in the saliva samples of OSCC patients. The levels of miR-145 in saliva samples of OSCC patients were higher in drinkers than in non-drinkers, suggesting its possible mediatory role in molecular pathways concerning alcohol consumption. There are currently no studies in the literature reporting an association between alcohol consumption and miR-145 expression in OSCC and in other types of cancer. However, there is evidence of modifiable lifestyle factors (including alcohol consumption) on plasma miRNA expression levels [71]. Notably, miRNA expression levels in plasma samples were low in all the three groups, and no statistically significant differences emerged in the expression levels of miRNAs in plasma. This result may suggest that liquid biopsy from saliva may be a more viable tool compared with plasma liquid biopsy for oral cancer, at least for these selected miRNAs, probably due to that saliva is taken from the anatomical location where the tumor developed.

There were some limitations that should be acknowledged for the proper interpretation of our results, including the limited sample size and the few miRNAs analyzed compared to those involved in oral carcinogenesis. Furthermore, it was not possible to evaluate the association between the considered miRNAs and the other risk factors for OSCC (i.e. HPV infection, hereditary syndromes (Fanconi’s anemia, dyskeratosis congenita), etc.), because none of our patients showed positivity to any of the abovementioned risk factors. This may be due to the small sample size, the low prevalence of HPV infection in oral cancer rather than oropharyngeal, and the low prevalence of hereditary syndromes [72].

In addition, it would be interesting in further studies to follow the evolution of the OSCCs and dysplastic OPMDs at different time points after the treatment, and compare the expression of salivary and plasmatic miRNAs with those in biopsy tissue.

Despite recent advances in the field of liquid biopsy, technical challenges remain, such as proper sample handling, the lack of a standardized protocol for the isolation and normalization of miRNAs, and regulatory issues, which limit its diffusion in clinical oral medicine practice [73].

## 5. Conclusions

Our results showed that liquid biopsy from saliva may be a useful and comfortable tool for the identification of diagnostic molecular biomarkers in OSCC and OPMD patients. In particular, circulating miR-138 and miR-424 in saliva samples emerged as early diagnostic biomarkers, discriminating well between OSCC patients, OPMD patients, and healthy subjects. 

Overall, although with some limitations remain to be addressed in larger studies, this work provided further evidence of the clinical relevance of circulating miRNAs as diagnostic biomarkers in oral oncology.

## Figures and Tables

**Table 1:** Demographic and clinical characteristics of the patients of the three groups.

Patient	Gender	Age	Smoker	Alcohol Consumer	Personal History of Cancer	Type of Lesions	Stage *	Localization	Treatment
**Group 1**									
1	M	64	yes	no	no	OSCC (G2)	1	AR	surgical resection
2	F	80	yes	no	yes	OSCC (G2)	4	BM	surgical resection + radiotherapy
3	M	73	no	no	no	OSCC (G2)	3	tongue	surgical resection + radiotherapy
4	M	55	no	no	no	OSCC (G2)	4	tongue	surgical resection + radiotherapy
5	F	61	yes	no	no	OSCC (G2)	4	tongue	surgical resection + radiotherapy
6	M	68	yes	yes	no	OSCC (G2)	3	FOM	surgical resection + radiotherapy
7	F	78	no	no	yes	OSCC (G2)	1	tongue	surgical resection
8	M	52	yes	yes	no	OSCC (G2)	4	tongue	surgical resection + radiotherapy
9	F	81	no	no	no	OSCC (G2)	3	tongue	surgical resection + radiotherapy
10	M	68	no	no	yes	OSCC (G2)	1	BM	surgical resection
11	F	65	no	no	no	OSCC (G2)	1	tongue	surgical resection
12	M	75	yes	no	no	OSCC (G2)	4	AR	surgical resection + radiotherapy
13	F	79	no	no	no	OSCC (G2)	4	tongue	surgical resection + radiotherapy
14	M	84	no	no	no	OSCC (G2)	2	AR	surgical resection
**Group 2**									
1	M	77	yes	no	yes	PVL with mild dysplasia	/	tongue, gingiva, hard palate, BM	laser ablation and follow-up
2	F	70	no	no	no	OLP with mild dysplasia	/	BM	topical corticosteroids
3	M	65	no	no	no	PVL with moderate dysplasia	/	hard palate and tongue	laser ablation and follow-up
4	F	58	yes	no	no	OLL with mild dysplasia	/	BM	surgical resection and removal of dental material
5	M	79	no	no	yes	OLP with mild dysplasia	/	BM and tongue	systemic corticosteroids
6	F	57	no	no	no	OLP with mild dysplasia		BM	topical corticosteroids
**Group 3**									
1	M	61	yes	no	no	Healthy control		/	/
2	F	72	no	no	no	Healthy control		/	/
3	F	48	yes	no	no	Healthy control		/	/
4	M	52	no	no	no	Healthy control		/	/
5	F	80	no	no	no	Healthy control		/	/

Legend: BM, buccal mucosa; FOM, floor of the mouth; AR, alveolar ridge. * 8th edition of the AJCC/UICC TNM staging system.

**Table 2:** Results of the ANOVA test for the miRNA expression levels from saliva samples among the three groups. Asterisk = the comparison between groups is significant for *p* ≤ 0.05.

Variable	Analysis of Variance; Statistical Significance: *p* ≤ 0.05							
	SS Effect	df Effect	MS Effect	SS Error	df Error	MS Error	F	*p*
**miR-21_s**	0.491583	2	0.245791	13.70107	22	0.622776	0.394671	0.678578
**miR-31_s**	0.043059	2	0.02153	4.02928	22	0.183149	0.117552	0.889649
miR-138_s *	0.538658	2	0.269329	1.0629	22	0.048313	5.574616	0.011001
**miR-145_s**	1.553145	2	0.776573	18.83423	22	0.856101	0.907104	0.418265
**miR-184_s**	0.035187	2	0.017594	0.15721	22	0.007146	2.4621	0.108412
miR-424_s *	0.046202	2	0.023101	0.14609	22	0.00664	3.478801	0.04867

**Table 3:** Results of the ANOVA test for the miRNA expression levels from plasma samples among the three groups.

Variable	Analysis of Variance; Statistical Significance: *p* ≤ 0.05							
	SS Effect	df Effect	MS Effect	SS Error	df Error	MS Error	F	*p*
**miR-21_b**	0.248505	2	0.124252	7.12042	22	0.323656	0.383903	0.685672
**miR-31_b**	0.000243	2	0.000121	0.00799	22	0.000363	0.33446	0.719301
**miR-138_b**	0.006047	2	0.003023	0.2178	22	0.0099	0.305389	0.73991
**miR-145_b**	0.995649	2	0.497824	28.46033	22	1.293651	0.384821	0.685064
**miR-184_b**	0.000011	2	0.000006	0.0007	22	0.000032	0.17839	0.837814
**miR-424_b**	1.12825	2	0.564125	32.49662	22	1.477119	0.381909	0.686995

**Table 4:** Results of the ANOVA test for the association between miRNA expression levels from saliva and plasma samples and smoking habits in OSCC patients. Asterisk = the association is significant for * *p* ≤ 0.05.

Variable	Analysis of Variance; Statistical Significance: *p* ≤ 0.05							
	SS Effect	df Effect	MS Effect	SS Error	df Error	MS Error	F	*p*
**miR-21_s**	0.775676	1	0.775676	3.04138	12	0.253448	3.060494	0.105722
**miR3-1_s**	0.198984	1	0.198984	0.86327	12	0.07194	2.765992	0.122164
**miR-138_s**	0.000929	1	0.000929	0.09657	12	0.008048	0.11545	0.739896
**miR-145_s**	0.579964	1	0.579964	13.23836	12	1.103197	0.525712	0.482315
**miR-184_s**	0.001046	1	0.001046	0.01674	12	0.001395	0.749777	0.403527
miR-424_s *	0.006783	1	0.006783	0.01576	12	0.001314	5.163991	0.042251
**miR-21_b**	0.886818	1	0.886818	6.22825	12	0.519021	1.708637	0.215655
**miR-31_b**	0.000552	1	0.000552	0.00663	12	0.000552	0.999868	0.33708
**miR-138_b**	0.026883	1	0.026883	0.1904	12	0.015867	1.694303	0.217469
**miR-145_b**	3.674721	1	3.674721	24.78074	12	2.065061	1.779473	0.206979
**miR-184_b**	0.00008	1	0.00008	0.00061	12	0.000051	1.579332	0.232769
**miR-424_b**	4.285125	1	4.285125	28.205	12	2.350416	1.823134	0.201857

**Table 5:** Results of the ANOVA test for the association between miRNA expression levels from saliva and plasma samples and drinking habits in OSCC patients. Asterisk = the association is significant for *p* ≤ 0.05.

Variable	Analysis of Variance; Statistical Significance: *p* ≤ 0.05							
	SS Effect	df Effect	MS Effect	SS Error	df Error	MS Error	F	*p*
**miR-21_s**	0.002989	1	0.002989	0.619293	12	0.051608	0.057913	0.813888
**miR-31_s**	0.00564	1	0.00564	0.266388	12	0.022199	0.254057	0.623359
**miR-138_s**	0.000683	1	0.000683	0.039585	12	0.003299	0.206913	0.657317
miR-145_s *	0.312089	1	0.312089	0.529068	12	0.044089	7.078613	0.02077
**miR-184_s**	0.000106	1	0.000106	0.00786	12	0.000655	0.161932	0.69446
**miR-424_s**	0.001124	1	0.001124	0.008707	12	0.000726	1.548489	0.237115
**miR-21_b**	0.014053	1	0.014053	0.632323	12	0.052694	0.266694	0.614942
**miR-31_b**	0.000092	1	0.000092	0.003164	12	0.000264	0.348916	0.565687
**miR-138_b**	0.001348	1	0.001348	0.073555	12	0.00613	0.219903	0.647519
**miR-145_b**	0.020575	1	0.020575	0.83404	12	0.069503	0.296022	0.596352
**miR-184_b**	0.000006	1	0.000006	0.000318	12	0.000027	0.229319	0.640637
**miR-424_b**	0.016404	1	0.016404	0.852331	12	0.071028	0.230951	0.639462

## Data Availability

The data presented in this study are available on request from the corresponding author due to privacy.
